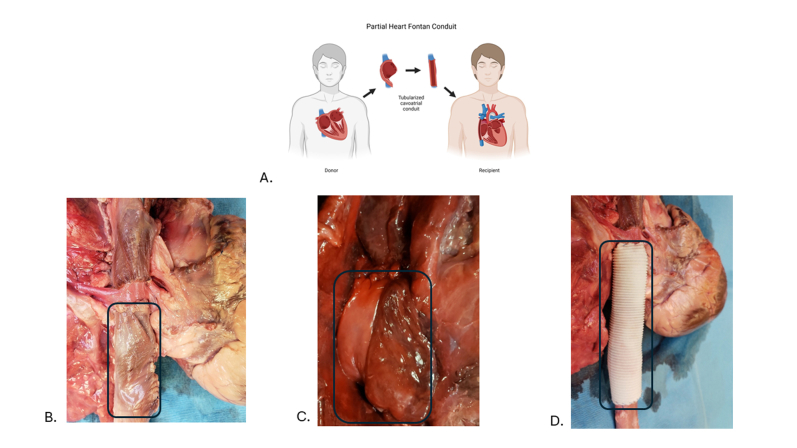# Cavoatrial Partial Heart Transplant: Ex Vivo Feasibility of a Novel Fontan Conduit

**DOI:** 10.1016/j.atssr.2025.08.017

**Published:** 2025-09-11

**Authors:** Harma Khachig Turbendian, Shannon Egli, Herra Javed, Anshaal Furrukh, Simon Chung, Taufiek Konrad Rajab

**Affiliations:** 1 Department of Pediatric and Congenital Heart Surgery, OSF Children's Hospital of Illinois, Peoria, Illinois; 2 Department of Surgery, University of Illinois College of Medicine - Peoria, Peoria, Illinois; 3 Department of Health Sciences Education - Anatomy, University of Illinois College of Medicine - Peoria, Peoria, Illinois; 4 Division of Pediatric Cardiovascular Surgery, Arkansas Children's Hospital, Little Rock, Arkansas

## Abstract

**Background:**

The Fontan circulation is the single common pathway for patients with single ventricle defects, but has adverse effects due to passive pulmonary blood flow. Pulsatile pulmonary blood flow may mitigate the adverse effects of Fontan circulation, but a subpulmonary assist device is yet to reach clinical use. We hypothesize that fresh right atrial homograft (cavoatrial partial heart transplant [CAPHT]) may be utilized as a pulsatile Fontan conduit with growth potential.

**Methods:**

The surgical technique for CAPHT conduit procurement was demonstrated in a live porcine donor. The CAPHT conduit was connected to a cardiopulmonary bypass circuit utilizing different cannulation orientations for inflow and outflow. Electrical activity and transduced pressure were recorded.

**Results:**

Freshly procured live porcine CAPHT conduits demonstrate pulsatility with extracorporeal perfusion. The conduits have a variable intrinsic rhythm but have the ability to be paced epicardially. With adequate volume loading, the average generated pulse pressure was 9.2 mm Hg. The CAPHT conduit maintains viability and pulsatility for at least 152 minutes while connected to extracorporeal perfusion.

**Conclusions:**

Our preliminary investigations demonstrate the technique for CAPHT conduit procurement. We show that the right atrium and vena cava can maintain viability pulsatility independent of coronary blood flow in an ex vivo setting. Subsequent in vivo studies will aim to evaluate the viability, function, and feasibility of the CAPHT conduit as an alternative extracardiac Fontan conduit that can be used as a subpulmonary assist device with growth potential.

**In Short**

▪ The cavoatrial partial heart transplant (CAPHT) conduit maintains viability in the absence of coronary arterial flow.
▪ CAPHT conduit is pulsatile with ex vivo perfusion.
▪ CAPHT can potentially be used as a subpulmonary assist device or an extracardiac Fontan conduit with growth potential.

Among patients living with a Fontan circulation, approximately 20%-30% experience signs and symptoms of Fontan failure.^1^^,^^2^ Extracardiac conduit undersizing (or outgrowth) can result in hepatic congestion, and conduit oversizing can lead to inefficient flow, stagnation, and thrombosis.^3^^,^^4^ One approach to address Fontan failure is to provide pulsatile pulmonary blood flow. Neither mechanical nor tissue engineering approaches have yet been translated into clinical practice for a subpulmonary Fontan assist device.

Partial heart transplantation uses living homografts to provide growing heart valve implants. We hypothesize that partial heart transplants using the right atrium can provide growing and pulsatile conduits for Fontan palliation. Here we demonstrate the technique for procurement of a cavoatrial partial heart transplant (CAPHT) conduit. We provide proof of principle that the conduit maintains pulsatility with extracorporeal support following procurement from a live porcine donor. The CAPHT conduit has the potential to be utilized as a pulsatile subpulmonary assist device or a growing extracardiac Fontan conduit.

## Material and Methods

Procurement of living CAPHT conduits was performed using adult Yorkshire (*sus domesticus*) pigs (Oak Hill Genetics) weighing 30-40 kg and approved by the local Institutional Animal Care and Use Committee protocol (IPROTO202300000160). Animal care conformed with the Principles of Laboratory Animal Care by the National Society for Medical Research and the Guide for the Care of Laboratory Animals by the National Institutes of Health. The Supplemental Video demonstrates the technique for CAPHT procurement, preparation, and perfusion. The heart was prepared for donation after midline sternotomy. After heparinization, activated clotting time >400 seconds was confirmed (ACT Plus Automated Coagulation Timer, Medtronic, cat no. ACT100). The pig was exsanguinated into a cardiopulmonary bypass circuit, which consisted of a multiflow cardiopulmonary bypass machine (LivaNova Stockert/Shiley CAPS), oxygenator (Capiox RX 05 Hollow Fiber Oxygenator, Terumo, cat no. CX∗RX05RE), quarter-inch tubing (Intersept, Medtronic, cat no. 228887339). Cardioplegic arrest was achieved with 15 mL/kg University of Wisconsin solution. The cardiectomy was performed, and the heart was maintained in ice cold University of Wisconsin solution. The donor heart was partitioned to provide the CAPHT conduit by separating the right atrium from the tricuspid annulus and atrial septum. The conduit was tubularized and connected to the cardiopulmonary bypass circuit inflow and outflow via the inferior vena cava, superior vena cava, and atrial appendage. Flow was maintained at normothermic temperature at 0.4-0.8 L/min. The electrocardiogram was obtained using epicardial pacing wires and pressure was recorded with a pressure transducer (Deltran, cat no. 6069). A Medtronic pacemaker (model 5388) was used for epicardial pacing. The electrocardiograms and blood pressures were recorded with BioAmp (FE 231) and BPAmp (FE 117) amplifiers and LabChart 8 software (AD Instruments). The video inset demonstrates the electrocardiogram and pressure tracings at the native rate and with epicardial pacing.

Planning operations for CAPHT were performed using anatomically normal fresh porcine and human cadaver hearts. Both donor and recipient porcine hearts were acquired from local vendors. Human cadavers were generously provided by the JUMP Simulation Center at OSF Healthcare. Bidirectional superior cavopulmonary shunts were performed in recipient hearts in standard fashion (Supplemental Figures B-D).

A linear mixed effects model was applied to the paced data, to evaluate the relationship between pressure difference as the outcome, and heart rate, orientation, and their interaction as fixed effects. Subject-specific random intercepts were included to account for repeated measures and intrasubject variability.

## Results

**Figure 1:**
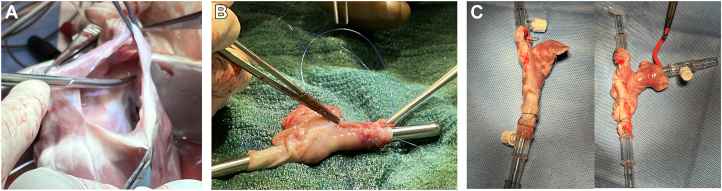
Surgical procurement and preparation of cavoatrial partial heart transplant (CAPHT) conduit for ex vivo perfusion. (A) Image of porcine heart after cardiectomy. An incision has been made in the right atrioventricular groove to initiate separation of the right atrium. (B) Right atrium and distal vena cava have been tubularized using 5-0 Prolene (Ethicon) suture over a Hegar dilator. (C) The CAPHT conduit cannulated for extracorporeal circulation in different orientations (left panel: caval connections; right panel: bicaval and atrial appendage connections).

CAPHT conduits were procured and perfused as described above using caval cannulation as part of a pilot ex vivo feasibility study (Figure 1; Supplemental Video). Viability was not assessed as part of this study, but extracorporeal perfusion and conduit pulsatility was maintained for a maximum of 152 minutes. The attempt was terminated due to circuit hemodilution and laboratory time/personnel constraints.

**Figure 2:**
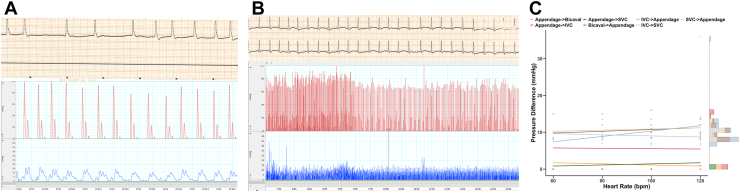
Rhythm and pulsatility of cavoatrial partial heart transplant (CAPHT) conduit with ex vivo perfusion. Representative electrocardiogram tracing (top panel) and pressure tracing (bottom panel; red: conduit outflow, blue: conduit inflow) at intrinsic rate (A) and paced rate of 120 bpm (B). (C) Pressure gradient measurements (mm Hg) across a range of paced heart rates (bpm) using different cannulation orientations for ex vivo perfusion. Each line represents the inflow-outflow pressure differential between specific cannulation orientations: inflow appendage, outflow bicaval; inflow appendage, outflow superior vena cava (SVC); inflow inferior vena cava (IVC), outflow appendage; inflow SVC, outflow appendage; inflow appendage, outflow IVC; inflow bicaval, outflow appendage; and inflow IVC, outflow SVC. Linear regression lines for each pathway are overlaid on individual data points, illustrating the trends in pressure gradients relative to paced heart rate.

Upon reperfusion, the intrinsic rate varied between 30 and 100 bpm (mean, 58 bpm). At these unpaced rates, the conduit generated a mean pulse pressure of 11.83 mm Hg (Figure 2A). The conduit could be paced epicardially, with a mean generated pulse pressure of 8.76 mm Hg (Figure 2B). The overall mean generated pulse pressure (paced and unpaced) was 9.2 mm Hg. Sequential iterations of the inflow/outflow cannulation strategy (inferior vena cava, superior vena cava, bicaval, and atrial appendage) were tested at varying heart rates to identify differences in the generated pulse pressure. These trends are demonstrated as linear regression plots in Figure 2C. Cannulation strategies that used the atrial appendage as outflow from the conduit generated statistically significant higher pulse pressures (10.5-11.4 mm Hg, *P* ≤ .001) than those that did not (Table 1). Regression slopes of pressure difference vs heart rate were not statistically significant for any pathways (*P* > .1) (Table 1). These findings suggest that the anatomic orientation of flow in the conduit had a greater influence than the heart rate on the generated pressure.

**Table 1:** CAPHT Pressure Generation as a Function of Flow Orientation and Pulse Rate

Inflow	Outflow	Mean Pressure Difference (mm Hg)	*P* Value	Pressure Difference/HR (mm Hg/bpm)	*P* Value	Unpaced HR (bpm)	n
Appendage	Bicaval	1.799 (-3.819, 7.416)	0.515	-0.015 (-0.201, 0.171)	0.872	<30	3
Appendage	IVC	6.165 (1.414, 10.916)	0.015	-0.005 (-0.136, 0.126)	0.939	114	1
Appendage	SVC	1.924 (-3.694, 7.541)	0.487	0.015 (-0.171, 0.201)	0.872	…	0
Bicaval	Appendage	11.196 (6.784, 15.609)	<0.001	0.024 (-0.076, 0.124)	0.631	46	4
IVC	Appendage	10.491 (6.165, 14.818)	0.001	0.073 (-0.018, 0.165)	0.115	52.5	4
IVC	SVC	9.688 (3.570, 15.805)	0.008	-0.015 (-0.146, 0.116)	0.82	70	3
SVC	Appendage	11.394 (6.967, 15.820)	<0.001	0.016 (-0.096, 0.127)	0.78	35	3

Values are presented as pressure (mm Hg) and heart rate (bpm).

CAPHT, cavoatrial partial heart transplant; HR, heart rate; IVC, inferior vena cava; SVC, superior vena cava.

## Comment

There is an estimated worldwide population of 50,000 to 70,000 patients with a Fontan circulation. The resultant physiology inevitably results in long-term complications as a result of passive, nonpulsatile pulmonary blood flow, inefficient hemodynamics, and static conduit size. Mechanical assist devices are an inadequate temporizing measure, and orthotopic heart transplantation remains the only definitive treatment. The evolution of the Fontan procedure included iterations that utilized the atrium as a source of contractile pulmonary blood flow.^5^^,^^6^ However, the procedures inherently reduced atrial size, introduced arrhythmic foci, risked native sinus node dysfunction, and were complex to perform.

Partial heart transplantation has altered the landscape of congenital heart surgery by providing a source of semilunar valve implants with the potential for growth and self-repair.^7^ We hypothesized that right atrial tissue could be procured and fashioned into a pulsatile conduit with growth potential. Orthotopic, biatrial heart transplant recipients’ right atrium maintains viability and pulsatility, as do portions of the right atrium when excised as either a patch or valve.^8^, ^9^, ^10^ Our ex vivo operation provides preliminary confirmation that neither the sinoatrial node nor coronary blood flow are necessary for right atrial pulsatility and viability. We have also shown that CAPHT conduits maintain an intrinsic rhythm and generate a pulse pressure for at least 2.5 hours with extracorporeal perfusion alone. Spatial flow orientation can modify the generated pressure, which reached a maximum of 11.4 mm Hg when the atrial appendage was positioned as the conduit outflow. We hypothesize that a pressure of 10-15 mm Hg would be sufficient to provide pulsatile flow at the time of initial Fontan procedure in an average single ventricle patient, and that a pressure of 15-25 mm Hg may be necessary for a patient with failing Fontan physiology and elevated pulmonary vascular resistance.

The donor and recipient techniques for CAPHT Fontan conduit are straightforward. The right atrium and cava are excised from a structurally normal donor heart and tubularized. This is then implanted as an extracardiac cavopulmonary conduit in a recipient heart (Supplemental Figure A-C). The procedure would have unique potential advantages. First, the conduit would be pulsatile and could serve as a subpulmonary assist pump. Second, the conduit would maintain growth potential just like the caval veins of heart transplants. Third, the living endothelial lining may render anticoagulation unnecessary. Disadvantages include the presumed need for immunosuppression, alloimmunization, donor scarcity, and logistical coordination for the procedure.

There are numerous avenues of investigation for future in vivo studies. This includes proving the long-term viability, maintenance of contractility, and growth of CAPHT conduits. We will have to demonstrate whether the circumferential pulsatility of an extracardiac conduit is superior to the unilateral pulsatility of a lateral tunnel Fontan. Progressive dilation is a concern, and external reinforcement with a vascular graft may mitigate this risk (Supplemental Figure D). Concomitant valve implantation may be necessary at the inferior vena caval and native bidirectional Glenn anastomoses in order to maintain antegrade pulmonary blood plow and prevent pulsatile flow into the native cavae. This could be achieved with donor pulmonary valve, bovine jugular vein, or bioprosthetic surgical / percutaneous pulmonary valves. Immunosuppression is an active area of investigation in partial heart transplantation and is a critical point when considering the feasibility of CAPHT conduits for either failing Fontan patients who are poor candidates for orthotopic heart transplantation, or as an alternative extracardiac conduit at the time of initial Fontan procedure.

In conclusion, this is proof of principle for a novel operation that proposes a pulsatile CAPHT conduit. This has the potential to be utilized as a subpulmonary assist device for failing Fontan physiology or as a growing extracardiac conduit.

## Figures and Tables

**Supplementary Figure:**