# Supplementary material for: Cavoatrial Partial Heart Transplant: Ex Vivo Feasibility of a Novel Fontan Conduit
Source: Ann Thorac Surg Short Rep. 2025 Sep 11;4(1):148–52. doi: 10.1016/j.atssr.2025.08.017 (PMC13100774; doi:10.1016/j.atssr.2025.08.017)
Supplement: Supplementary Material [file mmc2.docx]

Supplemental Figure: (A) Schematic representation of the proposed cavoatrial partial heart transplant Fontan procedure. A donor heart that is not usable for heart transplantation (e.g. due to ventricular dysfunction) is procured. The donor right atrium is dissected and fashioned into a pulsatile tube. This pulsatile tube is transplanted into the recipient to serve as a pulsatile Fontan conduit. Created in BioRender. Turbendian, H. (2024) BioRender.com/e77v358 cartoon (B) The CAPHT conduit (black outline) was procured from an anatomically normal pig heart, tubularzied, and implanted into a porcine recipient heart which had undergone a bidirectional Glenn. (C) This was repeated using a human cadaveric donor and bidirectional Glenn recipient. CAPHT conduit is outlined in black. (D) The vascular graft reinforced CAPHT conduit (black outline) is demonstrated implanted in a porcine bidirectional Glenn recipient.
